# Evaluation of the association of Wnt signaling with coronary artery calcification in patients on dialysis with severe secondary hyperparathyroidism

**DOI:** 10.1186/s12882-019-1543-3

**Published:** 2019-09-02

**Authors:** Tzung-Yo Ho, Nai-Ching Chen, Chih-Yang Hsu, Chien-Wei Huang, Po-Tsang Lee, Kang-Ju Chou, Hua-Chang Fang, Chien-Liang Chen

**Affiliations:** 1 Division of Nephrology, Kaohsiung Veterans General Hospital, No.386, Dazhong 1st Rd., Zuoying Dist, Kaohsiung City, 81362 Taiwan; 2 Department of Medicine, National Yang-Ming University School of Medicine, Taipei, Taiwan; 3 Department of Neurology, Kaohsiung Chang Gung Memorial Hospital, Chang Gung University College of Medicine, Kaohsiung, Taiwan

**Keywords:** Calcification, Secondary hyperparathyroidism, Bone mineral density

## Abstract

**Background:**

Patients with end-stage renal disease have a higher risk of death from cardiovascular events, which can be mainly attributed to coronary artery calcification (CAC). Wnt signaling is involved in vascular development and may play a role in vascular calcification. This study aimed to evaluate CAC prevalence in patients on dialysis with severe secondary hyperparathyroidism (SHPT) and identify CAC risk factors.

**Methods:**

The study is a retrospective analysis of the severe hyperparathyroidism registration study that prospectively recruited patients on dialysis with severe SHPT who were candidates for parathyroidectomy, from October 2013 to May 2015. CAC and bone mineral density (BMD) were measured. Demographic and clinical data including calcium, phosphorus, alkaline phosphatase, intact parathyroid hormone, Dickkopf-related protein 1 (DKK1), and sclerostin levels were analyzed. CAC scores were reported in Agatston units (AU).

**Results:**

A total of 61 patients were included in this study. No CAC, mild CAC (<100 AU), moderate CAC (>100 AU), and severe CAC (>400 AU) were observed in 4.9%, 11.4%, 14.8%, and 68.9% of patients, respectively. DKK1 and sclerostin were not associated with CAC. In univariate analysis, CAC was significantly correlated with age, sex (male), total cholesterol, and intravenous pulse calcitriol (*p*<0.05). CAC was not inversely correlated with the BMD, T scores, or Z scores of the femoral neck (*p*>0.05). In multivariate analysis, the stepwise forward multiple linear regression revealed that CAC was associated with age, male sex and intravenous pulse calcitriol (*p*<0.05). Furthermore, serum sclerostin was positively correlated with the BMD of the femoral neck but negatively associated with intact parathyroid hormone (*p*<0.05). Serum sclerostin was significantly associated with severely low bone mass with Z-scores<-2.5 of the femoral neck, even when adjusted for serum intact parathyroid hormone, vitamin D status, dialysis pattern, sex, and DKK-1 (*p*<0.05).

**Conclusions:**

The patients on dialysis with severe SHPT have a high prevalence of vascular calcification. Although the Wnt signaling pathway could play a role in hyperparathyroid bone disease, CAC may be mainly due to the treatment modality rather than the Wnt signaling pathway associated bone metabolism in patients on dialysis with severe SHPT.

**Electronic supplementary material:**

The online version of this article (10.1186/s12882-019-1543-3) contains supplementary material, which is available to authorized users.

## Background

The major cause of cardiovascular mortality in patients on dialysis with end-stage renal disease (ESRD) can be mainly attributed to vascular calcification [1, 2]. Patients with ESRD have a high prevalence of traditional coronary artery disease (CAD) risk factors such as hypertension and diabetes mellitus and non-traditional, uremia-related CAD risk factors, such as oxidative stress, inflammation and abnormal calcium-phosphorus metabolism [3–5]. Patients undergoing dialysis have mineral disturbances due to chronic kidney disease-mineral and bone disorders (CKD-MBD), which can result in altered bone structure and function. Furthermore, the rates of bone fractures are higher in patients undergoing dialysis compared with the general population [6, 7]. The severity of abdomen aortic calcification is associated with not only low bone mass, but also a high incidence of new fractures [8]. Results of the studies suggest a causal link between bone and vascular calcification. The calcium paradox is attributed to calcium shifting from bone to artery walls, resulting in bone softening and vessel hardening. Furthermore, the calcium paradox is likely caused by the differential responses of osteoblasts and osteoclasts to oxidative stress between the bone and artery. To date, many studies have highlighted the importance of the bone-vascular axis in the regulation of calcification between the two tissues [8–11].

The bone-vascular axis may work through a lot of signaling pathways. The Wnt signaling pathway plays a key role in bone resorption and formation. The Wnt signaling pathway, including Wnt proteins and their receptors, may have distinct roles in vascular development, too. Sclerostin and Dickkopf-1 (DKK1) are two soluble Wnt signaling inhibitors that bind to the LDL receptor-related proteins 5 and 6 co-receptors and inhibit the formation of active Wnt receptor complexes. DKK1 expression is altered in atherosclerotic plaques, thus suggesting a role for Wnt signaling in vascular calcification [12–14]. In addition, an increase in sclerostin expression is found in *in vitro* calcifying vascular smooth muscle cells studies [15]. These results suggest that the Wnt signaling pathway may provide a link between vascular calcification and bone.

CKD is associated with the development of secondary hyperparathyroidism (SHPT) and concomitant CKD-MBD. This can lead to an increased incidence of bone fractures and vascular calcification and, thus, higher rates of morbidity and mortality. There is growing evidence suggesting that the effects of parathyroid hormone (PTH) on bone may, at least partially, suppress the expression of sclerostin [16, 17]. Furthermore, the levels of serum sclerostin have been reported to negatively correlate with PTH levels [18]. These results suggest that the Wnt signaling pathway may provide a causal link between hyperparathyroidism, low bone mineral density, and possible vascular calcification via Wnt signaling pathway.

The American College of Cardiology/American Heart Association guideline updates 2019, taking into account the research results, places increased emphasis on the value of coronary artery calcification (CAC) scores for certain patient groups [19]. The current standard method of quantifying CAC is through ultrafast computed tomography (CT) scans, which reflect the extent of atherosclerosis measured by coronary angiography using Agatston units (AU) [20]. The other CT scoring method is the volume score as the sum of the total volume of calcium of the calcified regions in coronary artery vessels [21]. We hypothesized that the secreted Wnt antagonists sclerostin and DKK1 may play a role in the regulation of hyperparathyroid bone disease and in the process of vascular calcification in patients on dialysis with severe SHPT. The present study aimed to clarify the associations between circulating levels of sclerostin, DKK1, bone mineral density (BMD), and coronary artery calcification in patients on dialysis with severe SHPT and identify the risk factors for developing CAC.

## Methods

### Study protocol and subjects

The study was approved by the Institutional Review Board of Kaohsiung Veterans General Hospital, Kaohsiung, Taiwan (protocol title: Coronary calcification in dialysis patients with hyperparathyroidism: the contribution of traditional and uremia-related risk factors, VGHKS 15- CT11-01). The study is a retrospective analysis of the severe hyperparathyroidism registration study that prospectively recruited 61 patients on dialysis with severe SHPT who were candidates for parathyroidectomy from October 2013 to May 2015. This study included a select population of patients on dialysis with severe SHPT who had experienced clinical treatment failure (with poor control of hypercalcemia, hyperphosphatemia, bone pain, a history of serum iPTH level of >800 pg/mL), and were candidates for parathyroidectomy. All patients were at least 20 years old, had ESRD, and had been on maintenance hemodialysis or peritoneal dialysis. We exclude the patients with the following criteria: any unstable condition, malignancy, active inflammation/infection, pregnancy, lactating, and known alcohol or illicit drug abuse. Traditional coronary artery disease risk factors such as diabetes and hypertension and non-traditional, uremia-related CAD risk factors such as abnormal calcium-phosphorus metabolism were recorded. The SHPT treatment protocol consisted of two types of treatment modality: high-dose intravenous pulse calcitriol and low-dose oral calcitriol therapy. High-dose intravenous pulse calcitriol was administered intermittently at an initial dose of 2 μg twice weekly and increased, according to tolerance, up to a maximum dose of 2 μg thrice per week. On the other hand, oral calcitriol was administered at an initial dose of 0.5 μg and increased, according to tolerance, up to a maximum dose of 1 μg thrice per week. Bone mineral disorders were managed according to the treatment guidelines devised by the KDOQI [22].

The doses of calcitriol, phosphate binders, and dialysate calcium were titrated to maintain calcium × phosphate at ≤55 mg^2^/dL^2^, serum phosphate at ≤5.5 mg/dL and serum calcium at ≤10.5 mg/dL. All biochemical samples were obtained before the beginning of dialysis, including hemodialysis or peritoneal dialysis. Patients were treated with an appropriate peritoneal dialysate or hemodialysis dialysate containing a calcium concentration of 1.25 mmol/L or 1.5 mmol/L. All of the patients underwent routine hemodialysis three times a week (4 h per session) by standard high-flux dialysis or peritoneal dialysis. With regard to phosphate binders, calcium-containing compounds are preferred because of low cost. Intact parathyroid hormone (iPTH) was measured using an iPTH immunoradiometric assay kit (IRMA; Nichols Institute Diagnostics, San Diego, CA, USA) with a reference range of 12–65 pg/mL. Serum calcium, phosphorus, and alkaline phosphatase were measured using standard methodology. Serum samples for the analysis of DKK1 and sclerostin were aliquoted and stored at −80°C until use. Serum levels of DKK1 and sclerostin were measured in duplicate using ELISA kits (R&D Systems, Minneapolis, MN, USA). At the same time, reference mean ± standard deviation values for DKK-1 and sclerostin in 12 patients with end-stage renal disease with iPTH<500 pg/mL were 31.22±19.21 and 21.40±20.21 pmol/mL, respectively.

### Bone measurement

BMD was measured using a dual-energy X-ray absorptiometry scanner (Hologic Discovery DEXA Scanner; Hologic Inc., Bedford, MA, USA). Scans were made of lumbar vertebrae (L1–L4) and the proximal femur. Results are expressed as g/cm^2^. To overcome intra-patient measurement errors, we used the Hologic scanner, which automatically adjusts for body size and performed the procedure in a standard body position. Data were analyzed using Kaohsiung Veterans General Hospital in-hospital least significant parameters (spine = 0.022326 g/cm^2^; femoral neck = 0.026675 g/cm^2^), as customized in a previous study [23].

### Computed tomography (CT) data acquisition

All subjects underwent a synchronized multi-slice CT. Oral metoprolol (50-100) mg was administered before cardiac CT if the subject’s heart rate was higher than 65 beats per min. All scans were performed on a 64-slice multi-slice CT scanner (Aquilion 64; Toshiba Medical Systems Corporation, Otawara, Japan) to assess the CAC.

### Data evaluation

Kaohsiung Veterans General Hospital developed institutionally offline analysis of CT datasets to generate Agatston unit and volume scores. A calcified plaque was defined as an area of three connected pixels with a CT attenuation threshold of 130 Hounsfield Units (HU) [24]. The calcium volume score is calculated by multiplying the number of voxels with calcification by the volume. The Agatston unit was calculated by the weighted sum of calcified plaque lesions, multiplying the area of calcium by a factor (1 to 4) according to the method. [20]. CACs were calculated by adding the scores in the right coronary artery, the left anterior descending, left circumflex, and the left main coronary artery. The well-trained observers were blinded to clinical information and analyzed the coronary CT scans. We classified into four groups as following: CACs 0 HU (no calcification); mild calcification (1-100 HU); moderate calcification (101-400 HU), and severe calcification (>400 HU).

### Statistical analysis

All statistical analyses were performed with SPSS 13.0 for Windows. The data are expressed as mean ± standard deviation (SD) or as median plus interquartile range for skewed distributions. The outcomes are CAC, BMD, with Z-scores of the femoral neck. When the variables were not normally distributed or the relationship between the variables was not linear, the Spearman rank correlation test was used. Univariate and multivariate analyses of the factors associated with CAC scores were performed. Sex (male), age, dialysis, presence of diabetes mellitus, smoking history, hypertension, administration of intravenous calcitriol or lipid lower agents, Z-scores, T-scores and BMD of the femoral neck, calcium, phosphate, calcium x phosphate, alkaline phosphatase, iPTH, DKK1, and sclerostin were independent variables for CAC scores in univariate analysis. Multivariate analysis was performed using all of the possible variables (*p*<0.1). In addition, a Mann-Whitney U test was used to examine the continuous variable differences and a chi-square independence test was used to evaluate the differences in the two categorical variables, which were checked between patients with severely low bone mass and low bone mass based on Z-score of the femoral neck. *p*<0.05 was considered statistically significant. Binary logistic regression analyses were performed using all of the variables that were possible (*p*<0.1) to investigate the factors associated with severely low bone mass (Z score<-2.5).

## Results

A total of 61 patients with severe SHPT were referred to our outpatient department for parathyroidectomy. Due to pre-selection by medical doctors for surgery, no patients had any unstable condition, malignancy, active infection, pregnancy, were lactating, or had known alcohol or illicit drug abuse. There were no non-participating patients because all patients with stable condition were selected for parathyroidectomy. Causes of end-stage renal disease among the patients included chronic interstitial nephritis without renal biopsy (n=27), chronic glomerulonephropathy without biopsy (n=20), DM nephropathy (n=5), IgA nephropathy (n=4), and polycystic kidney disease (n=5). In Taiwan, calcium carbonate and calcium acetate are covered by health insurance as phosphate binders. Sevelamer and Fosrenol are self-paid. Only one patient had a history of non-calcium phosphate binders, Fosrenol or Sevelamer, in the preceding 2 months. However, due to them being too expensive, the patient could no longer afford them and stopped them.

The demographic and clinical data of the study group included baseline demographic data, DEXA measurement data, and CAC data (Table 1). All patients received bone scans which included four lumbar vertebrae (L1–L4) and the proximal femur. Only one patient’s lumbar spine data were omitted because of a previous spine operation. One patient’s Z-score of the femoral neck was omitted because they were 92 years of age and there were no reference values. High concentrations of bone metabolic markers (alkaline phosphatase = 219.44 ± 194.81 U/L; iPTH = 1194.50 ± 389.69 pg/mL) and low bone mineral content indicated bone mineral loss caused by high-turnover bone disease, resulting from SHPT. No CAC, mild CAC (<100 AU), moderate CAC (>100 AU), and severe CAC (>400 AU) were observed in 4.9%, 11.4%, 14.8%, and 68.9% of the patients, respectively. Aortic valve and mitral valve calcification were observed in 42.6% and 47.5% of the patients, respectively. We found significant lower serum sclerostin in dialysis patients with secondary hyperparathyroidism (Table 1; compared with the data of 12 patients with end-stage renal disease without hyperparathyroidism as laboratory internal control of sclerostin and DKK1, data not shown).

**Table 1:** Demographic and clinical data of the study group

	Secondary hyperparathyroidism (N = 61)	Reference value
Female/male	38/23	
Age (years)	57.11 ± 13.12	
Hemodialysis/peritoneal dialysis	57/4	
Calcium (mg/dL)	10.10 ± 0.80	8.4–10.6
Phosphate (mg/dL)	5.68 ± 1.56	2.1–4.7
Calcium x Phosphate (mg^2^/dL^2^)	57.51 ± 12.01	
Alkaline phosphatase (U/L)	219.44 ± 194.81	42–128
Intact parathyroid hormone (pg/mL)	1194.50 ± 389.69	8.0–76.0
DKK1(pmol/L)	41.38 ± 21.59	
Sclerostin (pmol/L)	7.76 ± 4.26	
Body weight (kg)	58.47 ± 10.42	
Dialysis duration (months)	93.37 (62.89,130.37)	
25(OH) Vitamin D (ng/mL)	27.94±12.0	>30
Albumin (g/dL)	3.85 ± 0.41	3.7-5.3
Total cholesterol (mg/dL)	179.07 ± 41.22	125-240
Triglyceride (mg/dL)	167.49± 151.33	20-200
BMD femoral neck (gm/cm^2^)	0.57 ± 0.15	
BMD lumbar spine (gm/cm^2^)	0.84 ± 0.18	
T-score of femoral neck	-2.83± 1.16	
Z-score of femoral neck	-1.43± 0.18	
Systolic blood pressure	143.75± 21.55	
Diastolic blood pressure	80.39±10.65	
Coronary artery (Agatston unit)	1570 (248.5,3701)	
Coronary artery (volume score)	1322 (226.5,3252.5)	
Aortic valve calcification	42.6%	
Mitral valve calcification	47.5%	
Intravenous pulse calcitriol	16.4%	
Diabetes mellitus	16.4%	
Hypertension	65.6%	
Smoking	1.6%	
Anti-hypertension drugs	65.6%	
Lipid lower agents	9.8%	

DKK1: Dickkopf-related protein 1, BMD: Bone mineral density

Mean± standard deviation or median (interquartile range)

As shown in Table 2, univariate correlations were significant between CAC scores and age, sex, total cholesterol, and intravenous pulse calcitriol (*p*<0.1). CAC scores did not inversely correlate with the BMD, T-scores, or Z-scores of the femoral neck (*p*>0.05). As shown in Table 3, multivariate regression analysis for CAC revealed that it was significantly associated with age, sex, and intravenous pulse calcitriol (*p*<0.05). Indeed, low cholesterol along with malnutrition-inflammation-atherosclerosis syndrome could be a factor in coronary artery calcification. We confirmed that intravenous pulse calcitriol independently explained increased coronary artery calcification, rather than low cholesterol. Univariate (Additional file 1: Table S1) and multivariate regression (Additional file 1: Table S2) analyses for CAC (volume score) showed that intravenous pulse calcitriol is the independent variable that explains increased coronary artery calcification, too.

**Table 2:** Univariate correlations between coronary artery calcification (Agatston unit) and parameters

Parameter	Correlation coefficient	*p*
Age (years) *	0.27	0.04
Dialysis duration (months)	0.18	0.16
Sex (female:0, male:1)*	0.47	<0.01
Body weight (kg)	0.16	0.22
Albumin (g/dL)	−0.24	0.07
Intact parathyroid hormone (pg/mL)	0.07	0.58
Total cholesterol (mg/dL)*	−0.38	<0.01
Triglyceride (mg/dL)	−0.09	0.50
Sclerostin (pmol//L)	−0.18	0.56
DKK1 (pmol//L)	−0.10	0.45
Calcium (mg/dL)	−0.16	0.21
Phosphate (mg/dL)	0.01	0.95
Calcium x Phosphate (mg^2^/dL^2^)	0.02	0.86
25(OH) Vitamin D (ng/mL)	0.14	0.29
Femoral neck (g/cm^2^)	-0.09	0.51
Lumbar spine g/cm^2^)	0.12	0.36
Systolic blood pressure	0.19	0.15
Diastolic blood pressure	0.09	0.49
Femoral neck T-score	−0.10	0.45
Femoral neck Z-score	0.10	0.47
Intravenous pulse calcitriol*	0.43	<0.01
Lipid lower agents	-0.03	0.79
Smoking	0.13	0.34
Diabetes mellitus	0.05	0.70
Hypertension	0.23	0.07

DKK1: Dickkopf-related protein 1, BMD: Bone mineral density

*p<0.05

**Table 3:** Output from forward stepwise regression analyses between multiple factors and coronary artery calcification (Agatston unit) in 61 dialysis patients

Variables	Beta	S.E	t statistics	*p*
Intravenous pulse calcitriol (yes: 1; negative : 0)*	2544.07	894.42	2.84	0.01
Sex (female:0; male:1)*	1688.01	681.54	2.48	0.02
Age (years)*	55.05	24.32	2.26	0.03
S.E: standard error				

*p<0.05

**Fig. 1:**
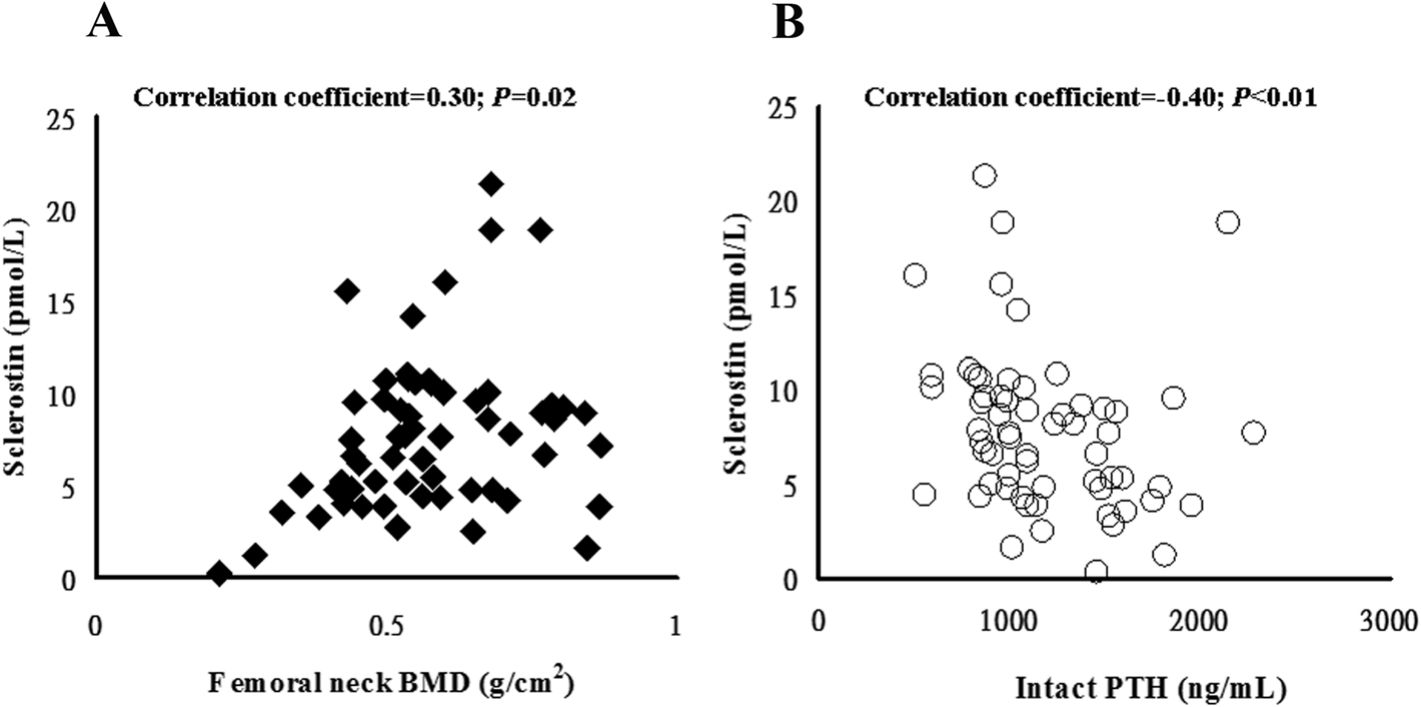
Nonparametric correlations of serum sclerostin with bone mineral density (BMD) of femoral neck and intact parathyroid hormone. Sclerostin positively correlated with (**a**) femoral neck BMD and negatively correlated with (**b**) intact parathyroid hormone

All possible factor correlations are presented for the femoral neck BMD or Z-scores alone instead of lumbar spine BMD, because abdominal aortic calcification led to a paradoxically raised BMD (Table 4). Correlation analyses were conducted to evaluate the relationship between serum sclerostin and serum DKK1 and iPTH. Serum sclerostin levels were significantly positively correlated with the femoral neck BMD (Fig. 1a), but negatively associated with intact parathyroid hormone (Fig. 1b; *p*<0.05). Demographic differences between patients with and without severe low bone mass are shown in Table 4. As shown in Table 5, binary logistic regression analysis for severely low bone mass (Z-score<-2.5) revealed that it was significantly associated with serum sclerostin (*p*<0.05), even when adjusted for hemodialysis, peritoneal dialysis, DKK1, vitamin D status, and parathyroid hormone. Sex is not included in the analysis because there were no male patients in the severely low bone mass groups. The results confirmed that sclerostin may play a role in hyperparathyroidism bone disease.

**Table 4:** Demographic differences between patients with severely low bone mass and low bone mass

Parameter	Severely low bone mass(Z-score≤-2.5) n=9	Low bone mass (Z-score>-2.5) n=51	*p*
Z-score of femoral neck	-2.9(-3.48,-2.68)	-1.4(-2,-0.25)	<0.01
BMD femoral neck (gm/cm^2^)	0.50 (0.30,0.54)	0.57 (0.50,0.68)	<0.01
Age (years)	46 (40, 66)	58 (49, 67)	0.22
Dialysis duration (months)	93.37 (69.25, 109.67)	91.97 (56.70, 130.60)	0.64
Sex (female/male)*	9/0	29/22	0.01
Hemodialysis/peritoneal dialysis*	7/2	49/2	0.04
Albumin (g/dL)	3.8 (3.15, 4.15)	3.9 (3.6, 4.10)	0.40
Intact parathyroid hormone (pg/mL)*	1527 (1074,1585)	1050 (874.60, 1380.90)	0.04
Sclerostin (pmol//L)*	3.84 (2.00, 7.60)	8.15 (5.14, 10.12)	<0.01
DKK1 (pmol//L)	24.71 (14.93, 48.14)	42.35 (25.31, 56.84)	0.09
25(OH) Vitamin D (ng/mL)*	19.05 (14.44, 27.58)	28.48 (20.60, 38.10)	0.02
Intravenous pulse calcitriol	22%	16%	0.63
Smoking	0%	2%	0.67
Diabetes mellitus	11%	18%	0.63
Hypertension	67%	65%	0.91
Lipid lower agents	0%	12%	0.28

*DKK1* Dickkopf-related protein 1, *BMD* Bone mineral density

median (interquartile range)

*p<0.05

**Table 5:** Binary logistic regression for multivariate analysis for the risk of increased prevalence of severely low bone mass (Z-score≤-2.5 of the femoral neck) in patients with severe hyperparathyroidism

Variables	Odds ratio	C.I.	Wald statistics	*p*
HD(1)/PD(0)*	177.41	(1.05-30101.24)	3.91	0.048
Sclerostin*	0.41	(0.20-0.86)	5.57	0.02
DKK1	0.89	(0.80-1.01)	3.43	0.06

*C.I*. 95% confidence interval, *HD* Hemodialysis, *PD* Peritoneal dialysis, *DKK1* Dickkopf-related protein 1

## Discussion

This study is the first to report associations between serum sclerostin, DKK1 levels, bone metabolic markers, bone mineral density and vascular calcification at the same time in patients on dialysis with severe SHPT. We found that the patients on dialysis with severe SHPT have a high prevalence of vascular calcification. Serum sclerostin was significantly positively correlated with the BMD of the femoral neck. Femoral neck BMD, T-scores, or Z-scores were not inversely correlated with CAC scores (*p*>0.05). DKK1 and sclerostin were also not associated with CAC scores (*p*>0.05). Although the Wnt signaling pathway could play a role in hyperparathyroid bone disease, CAC may not be linked to the Wnt signaling pathway that is associated with bone metabolism in patients with severe SHPT. However, the CAC was associated with age, male sex and intravenous pulse calcitriol (*p*<0.05).

Continued parathyroid hormone treatment decreases Dkk-1 mRNA levels in rat osteosarcoma cells in culture [25]. DKK1 is implicated in the pathogenesis of osteoporosis, which is reflected in increased DKK1 serum levels in postmenopausal women and the negative correlation between serum DKK1 and their BMD [26]. However, serum levels of DKK1 did not correlate with bone histomorphometric parameters and iPTH in patients with renal osteodystrophy [14]. Our data also indicate that serum levels of DKK1 were not associated with the bone parameters of patients on dialysis with severe SHPT. The lack of association between DKK1 and BMD may be attributed to the tissue distribution of DKK1 and the study group. The levels of sclerostin in the patients in our study were lower than those of patients without CKD in another study [18]. We found a statistically significant negative correlation between sclerostin and iPTH in this study, and this result agrees with the negative regulatory function of sclerostin in the transduction of PTH signals [16–18]. More importantly, we found that serum sclerostin was significantly positively correlated with the BMD of the femoral neck (Fig. 1a). An explanation could be that low sclerostin levels lead to increased osteoclast function and therefore increased bone resorption to reduce bone mass, while higher sclerostin levels lead to maintain bone mass by decreased bone resorption. Patients with more severe hyperparathyroidism have significantly lower sclerostin. Patients with higher sclerostin serum levels, and therefore less severe hyperparathyroidism, had a higher BMD as measured by DXA. Hence, sclerostin was positively associated with BMD in our patients with high bone turnover conditions, which is compatible with previous studies [27, 28]. Thus, the Wnt signaling pathway protein sclerostin seems to be associated with the pathogenesis of hyperparathyroid bone disease.

CAC is much more prevalent in patients with ESRD and contributes to high morbidity and mortality. Among patients with ESRD with severe SHPT in this study, we found that the prevalence of moderate to severe CAC (calcium scores higher than 400) was high (68.9%) in a southern Taiwan dialysis center. In contrast, the prevalence was 31% among patients without severe hyperparathyroidism [5]. Patient populations that differ in terms of SHPT appear to develop comparable degrees of CAC. A cross-sectional study of 205 patients on hemodialysis demonstrated that levels of serum phosphorus and calcium were positively correlated with the severity of CAC [29]. Furthermore, epidemiological studies have demonstrated that serum phosphorus and calcium × phosphorus are directly correlated with mortality, particularly with mortality from cardiovascular causes, in patients on hemodialysis [30, 31]. The levels of serum phosphate, calcium, and iPTH were high in this study; however, we found no relationship between CAC and these metabolic markers. It is possible that serum levels of calcium and phosphate may not represent the time-averaged calcium and phosphate levels and underestimated the overall calcium and phosphate positive balance. Furthermore, serum calcium should not be used as a guide to evaluate the true calcium balance because normal serum calcium concentrations do not preclude calcium positive balance [32, 33]. The increased calcium balance may not result in an increase in the serum calcium concentration, suggesting that calcium is being deposited in tissue.

In ESRD, a U-shaped bimodal response to bone turnover emerged with respect to vascular calcification. Indeed, in high bone turnover conditions with low serum sclerostin, calcium and phosphate could be released from the bone and contribute to vascular calcification. In low bone turnover conditions with high sclerostin, the patients exhibited the most extensive and severe arterial calcification [9]. We also evaluated the association of sclerostin and DKK1 with BMD and CAC. In theory, both sclerostin and DKK1 could have an effect on bone health and vascular disease, because of their associations with the mineralization process. Previous studies have demonstrated a negative correlation between vascular calcification and osteoporosis by evaluating the BMD of patients with normal kidney function and of those with uremia [34, 35]. However, we found no such correlations with CAC in the present study. Previous studies have revealed a negative association between circulating DKK1 and arterial stiffness in CKD patients [36] and calcified plaques in cases of type 2 diabetes mellitus [37]. In the present study, we found that DKK1, sclerostin, and BMD (of the femoral neck) were not associated with CAC. The bone metabolic factors seem to be masked by some factors. Multivariate regression analysis for CAC revealed that it was significantly associated with intravenous pulse calcitriol.

Vascular calcification could be prevented by endogenous calcification inhibitors such as klotho, osteopontin, fetulin-A and matrix Gla protein [38]. The effects of vitamin D on the vessel structure depend on physiological conditions or pharmacological effects. Vitamin D receptor activation can help protect against vascular calcification on physiological conditions. Low vitamin D doses leads to receptor activation in vascular smooth muscle cells, inhibiting matrix mineralization through by increasing calcification inhibitors klotho and osteopontin [39, 40]. However, a high dose of vitamin D could be associated with a 65 to 75% reduction in calcification inhibitor serum fetuin-A [41]. The therapeutic dose of 1, 25(OH)_2_D (usual dose at least 1000-fold higher than normal serum levels) is crucial for treating secondary hyperparathyroidism in patients with end-stage renal disease. 1, 25(OH)_2_D could directly increase matrix calcification of vascular smooth muscle cells *in vitro* [42]. Calcitriol accelerates vascular calcification irrespective of calcification inhibitor matrix Gla protein status [43]. A U-shaped curve relationship between vitamin D and vascular calcification was mentioned [44] and the hypothesis was confirmed by observations from children on dialysis in which both low and high levels of 1, 25(OH) _2_D were associated with vascular calcification [45]. Furthermore, administration of calcitriol could increases calcium and phosphate absorption. Using 1.5 to 1.75 mEq/L dialysate to suppress parathyroid hormone and calcium containing agents for phosphate-binding further increases positive calcium balance. The current thrice-weekly renal therapies including hemodialysis or peritoneal dialysis cannot completely remove the daily absorbed phosphate. People reach their peak bone mass around 30 years old and later lose bone mass with aging. This means that bone cannot absorb calcium and phosphate at older ages. Despite the use of 1.0 or 1.25 mmol/L calcium peritoneal dialysis solutions, the patients with calcium carbonate, and pulse alfacalcidol therapy are still associated with a positive calcium balance [46]. This huge positive calcium and phosphate mass transfer could lead to vascular calcification, especially in patients with oliguria. This may explain why CAC may be mainly attributable to treatment modality, such as large dose calcitriol. Large dose s of calcitriol skew the real calcium paradox by promoting vascular calcification due to its systemic effect through increased mineral burden and local effects on calcification inhibitors, which could mask the lesser amounts of calcium and phosphate released from the bone [47, 48].

The study has some limitations. The number of patients was rather small; however, to our knowledge, this is still the largest study on coronary artery and valvular calcification in patients with severe SHPT. Owing to its cross-sectional design, a causal relationship could not be determined. It is possible that low sclerostin is just a marker of hyperparathyroidism and plays no causative role. Furthermore, cytokine levels were measured only once at enrolment. The effects of time-averaged cytokine levels might be underestimated. In addition, the conclusions drawn from our data on patients on dialysis with severe SHPT should be treated with caution before extending them to other populations, such as groups with normal renal function and CKD without dialysis. The strength of this study is the determination of vascular calcification and valvular calcification by a 64-slice multi-slice CT scanner for simultaneous bone scan and precise calculation.

## Conclusions

Although the Wnt signaling pathway could play a role in bone metabolism during SHPT, CAC may be mainly attributable to treatment modality rather than bone metabolism in patients on dialysis with severe SHPT.

## Additional file

Additional file 1: **Table S1.** Univariate correlations between coronary artery calcification (volume score) and parameters. **Table S2.** Output from forward stepwise regression analyses between multiple factors and coronary artery calcification ion (volume score) in 61 dialysis patients. (DOCX 36 kb)

## Data Availability

The data that support the findings of this study are available from the Kaohsiung Veterans General Hospital, but restrictions apply to the availability of these data, which were used under license for the current study, and so are not publicly available. Data are available by contacting the corresponding authors upon reasonable request and with permission of the Kaohsiung Veterans General Hospital Research and Development Department.

## Abbreviations

AU: Agatston units
BMD: Bone mineral density
CAC: Coronary artery calcification
CAD: Coronary artery disease
CKD: Chronic kidney disease
CT: Computed tomography
DEXA: Dual energy X-ray absorptiometry
DKK1: Dickkopf-1
ESRD: End-stage renal disease
iPTH: Intact parathyroid hormone
SHPT: Secondary hyperparathyroidism
