# Supplementary material for: Evaluation of the association of Wnt signaling with coronary artery calcification in patients on dialysis with severe secondary hyperparathyroidism
Source: BMC Nephrol. 2019 Sep 2;20:345. doi: 10.1186/s12882-019-1543-3 (PMC6721332; doi:10.1186/s12882-019-1543-3)
Supplement: Supplementary file 1 — Table S1. Univariate correlations between coronary artery calcification (volume score) and parameters. Table S2. Output from forward stepwise regression analyses between multiple factors and coronary artery calcification ion (volume score) in 61 dialysis patients. (DOCX 36 kb) [file 12882_2019_1543_MOESM1_ESM.docx]

**Additional file 1**

**Table S1.** Univariate correlations between coronary artery calcification (volume score) and parameters.

| Parameter | Correlation Coefficient | *p* |
| --- | --- | --- |
| Age (years)* | 0.25 | 0.048 |
| Dialysis duration (months) | 0.20 | 0.13 |
| Sex(female:0, male:1)* | 0.45 | <0.01 |
| Body weight (kg) | 0.15 | 0.24 |
| Albumin (g/dL) | −0.22 | 0.09 |
| Intact parathyroid hormone (pg/mL) | 0.06 | 0.63 |
| Total cholesterol (mg/dL)* | −0.36 | 0.005 |
| Triglyceride (mg/dL) | −0.10 | 0.46 |
| Sclerostin (pmol//L) | −0.17 | 0.18 |
| DKK1 (pmol//L) | −0.10 | 0.43 |
| Calcium (mg/dL) | −0.18 | 0.16 |
| Phosphate (mg/dL) | 0.02 | 0.89 |
| Calcium x Phosphate (mg^2^/dL^2^) | 0.01 | 0.97 |
| 25(OH) Vitamin D (ng/mL) | 0.15 | 0.24 |
| Femoral neck (g/cm^2^) | -0.07 | 0.58 |
| Lumbar spine (g/cm^2^) | 0.14 | 0.30 |
| Intravenous pulse calcitriol* | 0.42 | 0.001 |
| Lipid lowering agents | -0.02 | 0.85 |
| Smoking | 0.13 | 0.31 |
| Diabetes Mellitus | 0.04 | 0.76 |
| Hypertension* | 0.26 | 0.045 |

**p*<0.05

**Table S2.** Output from forward stepwise regression analyses between multiple factors and coronary artery calcification ion (volume score) in 61 dialysis patients.

| Variables | Beta | S.E | t statistics | *p* |
| --- | --- | --- | --- | --- |
| Age(years)* | 49.62 | 18.37 | 2.70 | 0.01 |
| Sex(male : 1; female : 0)* | 1125.25 | 517.22 | 2.18 | 0.03 |
| Intravenous pulse calcitriol (yes: 1; negative : 0)* | 1896.15 | 671.35 | 2.82 | 0.01 |
| Duration(months)* | 10.22 | 4.14 | 2.47 | 0.02 |
| S.E: standard error |  |  |  |  |

*: mean P<0.05
